# CAREER DEVELOPMENT AWARDS AT NIA/NIH

**DOI:** 10.1093/geroni/igad104.0661

**Published:** 2023-12-21

**Authors:** Maria Carranza

**Affiliations:** National Institute on Aging, Bethesda, Maryland, United States

## Abstract

NIA offers a variety of individual research career development (K) awards, which enable scientists to enhance their careers in biomedical research. Mentored career development (K) awards are effective tools at retaining investigators in research careers and contributing to their subsequent research success. Specifically, those who receive K awards are significantly more likely to have subsequent research publications and to apply for subsequent NIH research awards. In this presentation, Dr. Carranza will review different K programs offered at NIA, including those targeted at postdocs, early career faculty, and more senior faculty, as well as clinician-science focused awards.

